# Autolysin Treatment of Cancer

**Published:** 1915-11

**Authors:** 


**Autolysin Treatment of Cancer.** Curran Pope of Louisville, Edw. Huntington Williams of Los Angeles, Hugh G. Nicholson of Charlestown, W. Va., and Andrew Wilson of Wheeling, separate articles in N. Y. Med. Jour., Oct. 9. The Horrowitz-Beebe extract is used, usually in the cellular tissue of the arm. Occasionally, intravenous injection is necessary. Considerable pain and caking follows the injection and usually a febrile reaction, the optimum being 100-100.4. Improvement may occur without reaction. Breaking down of superficial growths with increase in discharges occurs; in favorable cases, shrinkage of growth amounting to apparent cure in one or two cases. Cessation of odor is almost regularly noted and, usually gradual improvement so far as pain, cachexia, weight, appetite, blood, obstructive symptoms, etc., are concerned. Altogether, 48 cases are reported with generally favorable results in 32, unfavorable, or rather negative in 16. In the 16, we have included several in which improvement occurred but in which death followed. All the cases were inoperable.

